# Analysis of clinical and demographic characteristics of patients presenting with renal colic in the emergency department

**DOI:** 10.1186/1756-0500-1-79

**Published:** 2008-09-16

**Authors:** Mustafa Serinken, Ozgur Karcioglu, Ibrahim Turkcuer, Halis Ilke Ozkan, Mustafa Kemal Keysan, Aytaç Bukiran

**Affiliations:** 1 Emergency Physician, Department of Emergency Medicine, Pamukkale Univ, School of Medicine, Denizli, Turkey; 2 Emergency Physician, Department of Emergency Medicine, Bakirkoy Dr. Sadi Konuk Research and Training Hospital, Istanbul, Turkey

## Abstract

**Background:**

Renal colic (RC), is one of the most severe pain patterns which is most commonly diagnosed and managed in the emergency department (ED). This study is designed to evaluate the characteristics of adult patients presenting with pain and diagnosed with RC in the ED, length of stay in the ED and hospital and factors affecting these variables.

**Methods:**

All consecutive adult patients who presented with side pain, flank pain, abdominal or groin pain and consequently diagnosed with urolithiasis or RC were analyzed retrospectively. Sociodemographic data, times of admission into and discharge from the ED, adjunctive complaints, results of laboratory investigations, findings on examination, treatment and drugs administered were noted.

**Results:**

A total of 235 patients with a diagnostic code of urolithiasis were enrolled. Physicians were more likely to order radiological and laboratory investigations for female patients and those without hematuria in urinalysis. The peak incidence of patients diagnosed with RC (p = 0.001) was noted in August, while the winter had the lowest frequency of relevant admissions. The peak frequency was between 06:00 and 08:00. Women stayed longer in the ED (p = 0.001). Absence of hematuria in urinalysis was associated with increased length of stay (p = 0.007).

**Conclusion:**

Although RC is a common ED presentation for which the emergency physician has no guidelines in terms of diagnosis and management, there is no exact pattern to guide ordering investigations. Patients with atypical presentations stay longer in the ED and are likely to undergo additional tests in management.

## Background

Colicky pain originating from kidneys in flank area, namely renal colic (RC), is one of the most severe pain patterns which is commonly diagnosed and managed in the emergency department (ED). Severely increased intraluminal pressure following augmented blood flow with resultant ureteral smooth muscle contraction is accused in the mechanism of the pain. In addition, overt sensitivity of nociceptive receptors to mediators such as histamine and bradykinin are also implicated [1].

Most common manifestations of patients with renal colic in the ED are side pain or flank pain followed by abdominal pain, back pain, and groin pain [2]. Turkey is one of the countries with high incidence of urinary tract stone disease, thus a high frequency of patients with RC admitted in the EDs. While stone formation in the urinary tract affects about 5 to 10% of the population in most industrialized countries [3], the incidence of urinary stone disease in Turkey is estimated to be approximately 15% [4].

The evaluation and treatment of patients with nephrolithiasis is rapidly changing. New imaging modalities have emerged and have changed the way we evaluate patients with flank pain. Although a myriad of diagnostic tools including ultrasonography (USG), computed tomography (CT), kidney, ureter, and bladder radiograph (KUB) are useful aids in the diagnosis, clinical presumptive diagnosis generally direct treatment in the emergency setting.

This study evaluates the clinical and demographic characteristics of adult patients presenting with pain and diagnosed with RC in the ED, length of stay in ED and hospital and third factors affecting these variables.

## Methods

The present study was conducted in an industrialized middle-sized city of western Turkey, Denizli, in a University-based hospital. The hospital is one of the biggest hospitals in the city with an annual census of 24.000 patients. All consecutive adult patients presenting with side pain, flank pain, abdominal or groin pain and diagnosed with urolithiasis or RC by the emergency physicians between February 2005 and February 2007 were analyzed retrospectively. Patients with primary complaints other than pain such as nausea/vomiting, painless hematuria, etc. were excluded from the analysis. Also excluded were patients admitted into other healthcare institutions before being transferred to the study center with the consideration of confounding factors.

Patients younger than 15 years of age, patients with other complaints and those who had been previously diagnosed with RC and treated accordingly were excluded from the analysis. Detailed data relating to a total of 266 patients who received the code of urolithiasis (N20-23) in accord with International Classification of Diseases (ICD-10) were abstracted from the hospital information system.

Sociodemographic variables, times of admission and discharge from the ED, adjunctive complaints other than pain, results of laboratory and radiological investigations, findings on physical examination, treatment and drugs administered were noted and analyzed. Formally recorded entrance times were considered the starting point of stay in ED, and were calculated till discharge from ED or admission to the hospital wards.

Urinalysis was performed with IRIS iQ 200 Automated Urine Microscopic Analyzer (IRIS International, Inc. CA) in the hospital laboratory.

### Statistical analysis

All data obtained in the study were recorded and analyzed using the Statistical Package for Social Sciences for Windows, Version 11. Numerical variables were given as mean ± standard deviation (SD), and categorical variables were noted as N and percentages. Categorical variables regarding RC, length of stay, time of presentation and sociodemographic variables were compared to each other using chi-squared test. P values below .05 were considered statistically significant.

## Results

A total of 235 patients have received the diagnostic code of urolithiasis (N20-23) in accord with ICD-10 system and were considered eligible for the study. Male patients constituted 75.7% (n = 178) with a mean age of 31.7 ± 6.5 vs. a mean of 29.1 ± 8.2 for females. The ages of the samples averaged to 31.1 ± 7.0 years (range:16 and 66).

The presenting symptoms of the patients were side pain in 81.7% (n = 192), abdominal pain in 10.6% (n = 25), back pain in 5.9% (n = 14) and groin pain in 1.7% (n = 4).

Table 1 gives a list of common symptoms abstracted from the patients' charts with percentages. Side pain and flank pain were the two most common symptoms noted in both sexes (82.9% n = 195). Abdominal pain, cramps and spasms were encountered more frequently in female patients when compared to males (p = 0.001) (Table 1).

**Table 1:** List of the most common symptoms evaluated in both sexes.

**Symptoms**	**Total** n = 235 n (%)	**Male** n = 178 n (%)	**Female** n = 57 n (%)	P value
Side pain, flank pain	195 (82.9)	150 (84.2)	45 (78.9)	0.352
Abdominal pain, cramps, spasms	44 (18.7)	25 (14.0)	19 (33.3)	0.001
Pain radiation to the testicles or the vulvar area	32 (13.6)	28 (15.7)	4 (7.0)	0.950
Back pain, ache, soreness	20 (8.5)	14 (7.9)	6 (10.5)	0.586
Nausea and vomiting	20 (8.5)	13 (7.3)	7 (12.3)	0.276
Color change in urine	16 (6.8)	14 (7.9)	2 (3.5)	0.370
Painful urination	14 (5.9)	10 (5.6)	4 (7.0)	0.749
Fever	14 (7.2)	9 (5.0)	5 (8.8)	0.336
Groin pain	8 (3.4)	6 (3.4)	2 (3.5)	0.989
Urinary retention	7 (2.9)	6 (3.4)	1 (1.7)	0.981

Table 2 shows that flank tenderness (FT) is the most common finding in the physical examination of these patients (58.7% n = 138). The table depicts an analysis of laboratory test results with regard to sex, presence of hematuria, FT and lower quadrant tenderness. An urinalysis was ordered for all patients while complete blood count was obtained in 129 (54.9%) and USG in 43 (18.3%) (Table 2). No radiological test was ordered in a majority of the patients (n = 170, 72.3%).

**Table 2:** Analysis of laboratory test results with regard to sex, presence of hematuria, FT, and lower quadrant tenderness.

	**CBC** n (%) 129 (54.9)	**Biochemical tests **(creatinine, electrolytes vb.) n (%) 62 (26.4)	**KUB** n (%) 54 (23.0)	**USG** n (%) 43 (18.3)	**BT** n (%) 31 (13.2)
**Sex**					
Female (n = 57)	44 (77.1)	18 (31.5)	16 (28.1)	16 (28.1)	8 (14.0)
Male (n = 178)	85 (47.7)	44 (24.7)	38 (21.3)	27 (15.1)	23 (12.9)
*P value*	*0,001*	*0.306*	*0.294*	*0.028*	*0.829*
Hematuria*					
No (n = 41)	38 (92.7)	32(78.0)	12 (29.2)	25 (60.1)	16 (39.0)
Yes (n = 194)	91 (46.9)	30 (15.4)	42 (21.6)	18 (9.2)	15 (7.7)
*P value*	*0.001*	*0.001*	*0.292*	*0.001*	*0.001*
**Flank Tenderness**					
No (n = 97)	57 (58.7)	25 (25.7)	21 (21.6)	26 (26.8)	14 (14.4)
Yes (n = 138)	72 (52.1)	37 (26.8)	33 (23.9)	17 (12.3)	17 (12.3)
*P value*	*0.318*	*0.859*	*0.685*	*0.005*	*0.637*
**Lower Quadrant Tenderness**					
No (n = 193)	94 (48.7)	46 (23.8)	45 (23.3)	36 (18.6)	24 (12.4)
Yes (n = 42)	35 (83.3)	16 (38.1)	9 (21.4)	7 (16.6)	7 (16.6)
*P value*	*0.001*	*0.057*	*0.792*	*0.763*	*0.463*

* 5 and more RBC/HPF on a centrifuged urine specimen

Physicians were found to be more likely to order radiological and laboratory investigations for female patients and those without hematuria (<5 RBC/HPF) in urinalysis (Table 2). Intramuscular administration of nonsteroidal anti-inflammatory drugs (NSAIDs) was the most common treatment undertaken in the ED (86.4%) (Table 3). On the other hand, 54 (23.0%) patients received a treatment composed of IM NSAIDs together with IV opiates and IV hydration. Meanwhile, 22 (9.4%) patients were not treated with NSAIDs and received only IV opiates and IV hydration. Diclofenac sodium was the most commonly chosen NSAID administered via IM route (n = 195, 96%) while meperidine was the most common opiate preferred in the sample (n = 72, 94.7%).

**Table 3:** Distribution of treatment and drugs administered to the patients with RC.

**Treatment and drugs**	**N**	**%**
IM NSAIDs	203	86.4
IV line started	165	70.2
IV hydration*	149	63.4
IV Opiates	76	32.3
IV antiemetics	37	15.7
IV NSAIDs	16	6.8

* 250 ml or more normal saline or lactated Ringer's solution.

Only 36 (15.3%) patients were consulted with other disciplines and more than four-fifth of this subgroup (n = 28) were hospitalized with the resultant hospitalization rate of 11.9% for the whole sample. Of those admitted to the wards, 17 had pain or vomiting despite analgesics and antiemetics, whereas 13 (5.5%) suffered from infected hydronephrosis. Mean length of stay in hospital was 4.2 ± 1.9 days.

As far as the patterns and times of presentations to the ED, August had the peaked rate of patients diagnosed with RC (p = 0.001) (Table 4). Winter had the lowest frequency of admissions with RC (Table 4). The day with the lowest frequency was found to be Sunday (p = 0.046) (Table 4). The peak frequency was between 06:00 and 08:00 with more than half (51,0%) of all admissions between 04:00 and 12:00 (Table 5).

**Table 4:** Distribution of patients with RC with respect to the time of admission in the ED.

	**# of patients ** presented to ED	**Patients diagnosed ** **with RC n (%)**	**Percentage of the ** **diagnosis(/thousand)**	P value
**Month**				
January	2027	16	7.9	0.001
February	1606	10	6.2	
March	1767	25	14.1	
April	1660	13	7.8	
May	1866	12	6.4	
June	1780	23	12.9	
July	2371	29	12.2	
August	2068	35	16.9	
September	1667	19	11.4	
October	1977	29	14.6	
November	1943	13	6.7	
December	2208	11	4.9	
**Seasons**				
Winter	5841	37	6.3	0.001
Spring	5293	50	9.4	
Summer	6219	87	14.0	
Autumn	5587	61	10.9	
**Days of the week**				
Monday	3401	34	10.0	0.046
Tuesday	2916	43	14.7	
Wednesday	3280	31	9.4	
Thursday	3302	42	12.7	
Friday	3722	37	9.9	
Saturday	3110	26	8.4	
Sunday	3209	22	6.8	
**Total**	**22940**	**235**		

**Table 5:** Time of presentation of patients in the ED.

**Time of presentation (hrs)**	**n**	**%**
00:00 – 01:59	7	3.0
02:00 – 03:59	4	1.7
04:00 – 05:59	13	5.5
06:00 – 07:59	39	16.6
08:00 – 09:59	32	13.6
10:00 – 11:59	36	15.3
12:00 – 13:59	22	9.3
14:00 – 15:59	20	8.5
16:00 – 17:59	15	6.4
18:00 – 19:59	17	7.2
20:00 – 21:59	19	8.0
22:00 – 23:59	11	4.7

Mean length of stay in ED was found to be 87.6 min (± 42.4 min) (range: 41 and 487 min). Seventy-eight patients (33.2%) stayed in ED for longer than two hours, 101 (43.0%) patients for 1 to 2 hours, while 56 (23.8%) stayed for shorter than one hour. Female patients tended to stay longer in the ED when compared to males (p = 0.001) (Table 6).

**Table 6:** Length of stay in ED and analysis of factors affecting these variables.

		**Length of stay in ED**			
			
	n (%)	**<1 h** n (%)	**1–2 h** n (%)	**>2 h** n (%)	P value
**Sex**					
Male	178 (75.7)	49 (27.5)	82 (46.0)	47 (26.4)	0.001
Female	57 (24.3)	7 (12.3)	19 (33.3)	31 (54.4)	
**Age**					
16–25	50 (21.2)	13 (26.0)	20 (40.0)	17 (34.0)	0.990
26–35	126 (53.6)	29 (23.0)	55 (43.6)	42 (33.3)	
>35	59 (25.2)	14 (23.7)	26 (44.0)	19 (32.2)	
RBC in urinalysis*					
>20	116 (49.4)	33 (28.4)	51 (44.0)	32 (27.6)	0.007
5–20	78 (33.2)	20 (25.6)	35 (44.9)	23 (29.5)	
<5	41 (17.4)	3 (7.3)	15 (36.6)	23 (56.1)	
WBC in urinalysis*					
>5	44 (18.7)	10 (22.7)	21 (47.7)	13 (29.5)	0.768
≤ 5	191 (81.3)	46 (24.1)	80 (41.9)	65 (34.0)	
**Flank tenderness**					
Yes	138 (58.7)	40 (29.0)	72 (52.1)	26 (18.8)	0.001
No/unknown	97 (41.3)	16 (16.5)	29 (29.9)	52 (53.6)	
**Lower quadrant tenderness**					
Yes	42 (17.9)	8 (19.0)	10 (23.8)	24 (57.1)	0.001
No	193 (82.1)	48 (24.9)	91 (47.1)	54 (28.0)	

* HPF on a centrifuged specimen

Table 6 depicts the length of stay in ED and factors affecting these variables. Absence of hematuria in urinalysis was associated with increased length of stay (p = 0.007). Detection of FT on examination was associated with shortened length of stay in ED (p = 0.001).

## Discussion

ED is the typical health facility to receive patients with urolithiasis and/or RC worldwide. It was reported that male-to-female ratio was 3 with a peak onset of symptomatic nephrolithiasis in the third and fourth decades of life [5,6]. The results of the present study gave similar figures (male-to-female ratio 3:1 and mean age 31.1 ± 7.0).

Side pain or flank pain was reported to be the symptom which was most commonly complained of by patients with RC (37.5%) followed by abdominal pain (16.2%) [5]. Likewise, these symptoms ranked high among others noted in the present study (84.3%, 21.7%, respectively).

Our results indicate that female patients with RC were more likely to stay in ED longer, to go to radiology suit and to have their blood drawn for laboratory tests when compared to males. Abdominal complaints were also noted more frequently in females. It can be concluded that emergency physicians tend to order more tests to consider a clinical diagnosis of RC in females possibly due to a broad list of differential diagnoses in females.

The most common finding in patients with ureterolithiasis is FT due to the dilation and spasm of the ureter from transient obstruction as the stone passes from the kidney to the bladder [5]. The present results also demonstrated that FT was remarkable in a majority of patients diagnosed with RC (84.3%). Absence of FT was found to be associated with increased length of stay in ED and more frequent orders for USG when compared to patients with FT. This result suggests that absence of FT leads the physician to revise the presumptions and consider alternative diagnoses such as abdominal catastrophes and to "err on the patient's side", via ordering more tests.

The urinalysis is the investigation most commonly ordered in patients with presumptive diagnosis of RC and reveals hematuria in 90% of patients with stones. On the other hand, one-tenth of patients with RC would not exhibit hematuria [7]. One retrospective study found that 67% of patients with ureterolithiasis had more than 5 RBC per HPF and 89% of patients had more than zero RBC/HPF on microscopic examination of the urine [8]. Hematuria was recorded in 82,5% (n = 194) in the present sample. Absence of hematuria was associated with increased length of stay in ED and more likelihood of laboratory and radiological investigations, also suggesting a difficulty in diagnosis in presence of a sample of urine devoid of RBCs. Similarly, absence of lower quadrant tenderness was associated with elongated length of stay, along with increased rate of ordered investigations. It can be speculated that the physicians are urged to reconsider the worst or alternative scenarios (appendicitis, diverticulitis, tuboovarian diseases, ectopic pregnancy, ischemic bowel disease, pyelonephritis, abdominal aortic aneurysm, renal artery thrombosis, etc.) in the absence of tenderness in the lower abdomen. Some patients who had been recommended for admission to the hospital wards were left in the ED longer than expected due to some reasons related to disciplines involved. These affected the mean length of stay.

Admission times in the current study suggest that the weather affects the frequency of referrals with RC. This is due to dehydration in hot weather from increased perspiration resulting in concentrated and acidified urine. These changes could lead to crystallization with a higher likelihood to form stones. There are concrete data reporting higher incidence of admissions due to RC in hotter climates and in summer [7,9]. There are also papers reporting that increased intake of fluids helps reduce stone formation [10-12].

The current data also showed that most patients visited the ED in the morning hours. According to anecdotal evidence people are more at risk of renal colic during the night. Nonetheless, recent data suggested a circadian variability for the occurrence of renal colic, with a pattern characterized by a morning peak, independent of gender and presence or not of demonstrable kidney stones [13,14]. Manfredini et al. noted that episodes of renal colic show a significant circadian pattern, with a morning peak and a nadir in the afternoon [13]. Urine production and renal excretion rates of solutes rise during daytime and reach minimum values at night. Also, the glomerular filtration rate exhibits a circadian rhythm peaking in the morning [15]. Studies on healthy people and people who have had kidney stones showed a higher risk of calcium oxalate crystallization in the morning [16].

The data derived from the present sample showed the lowest incidence of admissions with RC on Sundays, which is difficult to interpret. Engagement with other activities and/or consumption of larger amounts of liquids might be responsible for the decreased rate of referral for holiday makers.

When faced with the patient who has symptoms consistent with acute RC, the physician must decide if the patient needs emergent imaging. It can be discussed that the physicians underutilized the radiological investigations to diagnose RC in the current study when compared to literature data.

CT is generally the preferred imaging modality to diagnose renal colic. Ultrasound is preferred in pregnant women to minimize radiation exposure in the western world [17]. Brown conducted a US-based study and pointed out that 25% of cases with RC in ED underwent CT, 5% underwent USG while no radiological study was needed in 53% [2]. In the present study the percentage diagnosed without a radiological test was higher than literature data (72%) as well as a higher rate of USG orders (18%) but a lower incidence of CT orders (13%). Lesser frequency of CT orders can be attributed to cost effectiveness issues in a developing country with a very low income per capita.

## Conclusion

Although RC is a common ED presentation for which the emergency physician has no guidelines in terms of diagnosis and management. Physicians usually perceive presumptive diagnoses sufficient to start treatment. There is no exact pattern to guide the art of ordering laboratory and radiological investigations, with resultant variations in approach to patients. Patients with atypical presentation patterns stay longer in the ED and are likely to undergo additional tests in management.

## References

[B1] Perlmutter A, Miller L, Trimble LA, Marion DN, Vaughan ED, Felsen D (1993). Toradol, an NSAID used for renal colic, decreases renal perfusion and ureteral pressure in a canine model of unilateral ureteral obstruction. J Urol.

[B2] Brown J (2006). Diagnostic and treatment patterns for renal colic in US emergency departments. Int Urol Nephrol.

[B3] Bartoletti R, Cai T, Mondaini N, Melone F, Travaglini F, Carini M, Rizzo M (2007). Epidemiology and risk factors in urolithiasis. Urol Int.

[B4] Akinci M, Esen T, Tellaloglu S (1991). Urinary stone disease in Turkey: an updated epidemiological study. Eur Urol.

[B5] Craig S Renal Calculi. Emergency Medicine web site. http://www.emedicine.com/emerg/topic499.htm.

[B6] Menon M, Parulkar BG, Drach GW, Walsh PC, Retik AB, Vaughan ED, Wein AJ (1998). Urinary lithiasis: Etiology, diagnosis, and medical management. Campbell's Urology.

[B7] Manthey DE, Teichman J (2001). Nephrolithiasis. Emerg Med Clin North Am.

[B8] Bove P, Kaplan D, Dalrymple N, Rosenfield AT, Verga M, Anderson K, Smith RC (1999). Reexamining the value of hematuria testing in patients with acute flank pain. J Urol.

[B9] Stoller ML, Bolton DM, Tanagho EA, McAnninch JW (1995). Urinary stone disease. Smith's General Urology.

[B10] Jenkins AD, Gillenwater JY, Grayback JT, Howards SS, Duckett JW (1996). Calculus formation. Adult and Pediatric Urology.

[B11] Finlayson B, Smith A (1974). Stability of first dissociable proton of uric acid. J Chem Eng Data.

[B12] Ngo TC, Assimos DG (2007). Uric Acid nephrolithiasis: recent progress and future directions. Rev Urol.

[B13] Manfredini R, Gallerani M, Cecilia O, Boari B, Fersini C, Portaluppi F (2002). Circadian pattern in occurrence of renal colic in an emergency department: analysis of patients' notes. BMJ.

[B14] Boari B, Manfredini R (2003). Circadian rhythm and renal colic. Recenti Prog Med.

[B15] Koopman MG, Koomen GC, Krediet RT, de Moor EA, Hoek FJ, Arisz L (1989). Circadian rhythm of glomerular filtration rate in normal individuals. Clin Sci (Lond).

[B16] Singh RK, Bansal A, Bansal SK, Singh AH, Mahdi AA (1993). Circadian periodicity of urinary inhibitor of calcium oxalate crystallization in healthy Indians and renal stone formers. Eur Urol.

[B17] Engineer R, Peacock WF, Tintinalli JE, Kelen GD, Stapczynski JS (2004). Urologic Stone Disease. Emergency Medicine: A Comprehensive Study Guide.

